# Artesunate Alleviates Paclitaxel-Induced Neuropathic Pain in Mice by Decreasing Metabotropic Glutamate Receptor 5 Activity and Neuroinflammation in Primary Sensory Neurons

**DOI:** 10.3389/fnmol.2022.902572

**Published:** 2022-05-27

**Authors:** Yize Li, Jiamin Kang, Ying Xu, Nan Li, Yang Jiao, Chenxu Wang, Chunyan Wang, Guolin Wang, Yonghao Yu, Jingjing Yuan, Linlin Zhang

**Affiliations:** ^1^Department of Anesthesiology, Tianjin Research Institute of Anesthesiology, Tianjin Medical University General Hospital, Tianjin, China; ^2^Department of Anesthesiology, Pain and Perioperative Medicine, The First Affiliated Hospital of Zhengzhou University, Zhengzhou, China

**Keywords:** artesunate, mGluR5, neuroinflammation, paclitaxel, chemotherapy-induced peripheral neurotoxicity, neuropathic pain

## Abstract

Experimental studies on the pathogenetic process of paclitaxel-induced neuropathic pain (PINP) have been initially carried out, but PINP still has no effective therapy. Recently reported studies have highlighted the involvement of glutamate receptors and neuroinflammation in peripheral and central nociceptive transmission in PINP. Artesunate is a first-line antimalarial drug with established efficacy in alleviating pain in a variety of pathologies. The current work assessed whether artesunate inhibits PINP by modulating metabotropic glutamate receptor 5 (mGluR5) and neuroinflammation in mice. The anti-hyperalgesic effect of artesunate was verified by assessing mechanical frequency and thermal latency in the paw withdrawal test as well as spontaneous pain. The expression levels of mGluR5, pain-related receptors and neuroinflammatory markers in dorsal root ganglion (DRG) were examined. In addition, treatment with CHPG and 2-methyl-6-(phenyl ethynyl) pyridine (MPEP) (mGluR5 agonist and antagonist, respectively) was performed to determine mGluR5’s role in the anti-hyperalgesic properties of artesunate. We demonstrated artesunate prevented PINP in a dose-dependent manner, while exerting a clear anti-hyperalgesic effect on already existing PINP. Artesunate normalized paclitaxel-related expression changes in DRG mGluR5, NR1, and GluA2, as well as six paclitaxel related neuroinflammation markers. Intrathecal application of MPEP treated PINP by reversing NR1 and GluA2 expression changes but had no effects on chemokines and inflammatory factors. Furthermore, artesunate treatment reversed acute pain following CHPG application. In conclusion, this study revealed that artesunate alleviates paclitaxel-induced hyperalgesia and spontaneous pain by decreasing DRG mGluR5 expression and neuroinflammation in the mouse model of PINP.

## Highlights

- Artesunate reduces paclitaxel-induced NP and mGluR5 expression.
- Artesunate reduces chemotherapy-induced neuroinflammation.
- Metabotropic glutamate receptor 5 (mGluR5) antagonist prevents chemotherapy-induced neuropathic pain.
- Artesunate impairs mGluR5 agonist-evoked acute pain.

## Introduction

Paclitaxel isan antimicrotubule drug utilized for diverse malignancies, including breast, ovarian, lung and prostate cancers (Weaver, 2014). Chemotherapy-induced peripheral neurotoxicity (CIPN) represents a commonly detected secondary effect of taxane therapy, with up to 87% of treated cases likely developing peripheral neurotoxicity (Banach et al., 2017). Taxane therapy often causes distal axonal neuropathy; specifically, numbness and tingling constitute the commonest undesirable effects of paclitaxel-induced peripheral neurotoxicity (Zhang et al., 2016; Klein and Lehmann, 2021; Timmins et al., 2021) and occur at high taxane doses (Argyriou et al., 2008). Paclitaxel treatment has also been linked to acute pain syndrome, which produces myalgia and arthralgia. Paclitaxel-induced acute pain syndrome occurs in up to 70% of patients and tends to peak 3 days after paclitaxel treatment, and the associated symptoms generally subside within 7 days (Loprinzi et al., 2011). In patients administered paclitaxel, up to 50% may still exhibit lower extremity sensory disturbances 12 months after treatment (Timmins et al., 2021), and the symptoms persist for a long time (Pachman et al., 2016). This affects long-term function and the quality of life in individuals surviving cancer. Currently, dose reduction is the only approach for preventing serious CIPN, and clinicians must make tough decisions to balance maximal therapeutic dose with minimal long-term undesirable effects. Despite the well-established potent anticancer effects of paclitaxel, the specific molecules involved in the pathogenetic process of paclitaxel-induced neuropathic pain (PINP) remain unclear, indicating the importance of developing effective means for preventing and treating CIPN (Cavaletti and Marmiroli, 2018).

Artesunate (ART) represents a semisynthetic water-soluble derivative of artemisinin, which is produced by the traditional Chinese medicinal plant *Artemisia annua* and was first developed for malaria treatment. Additionally, artesunate has significant antitumor properties, induces DNA damage and inhibits DNA damage repair (Zhao et al., 2020). Recent advances have deepened our understanding of artemisinin, which alleviates oxidation (Tsuda et al., 2018), immune responses (Bang et al., 2021) and inflammation (Yang et al., 2012; Feng and Qiu, 2018; Yin et al., 2021). Artesunate currently remains the first-line antimalarial medicine, with definite efficacy and low toxicity (Zou et al., 2020). Most importantly, artesunate was shown to attenuate complete Freund’s adjuvant (CFA)-induced acute inflammatory pain (Guruprasad et al., 2015) and remifentanil-induced incisional hyperalgesia in rodents (Zhang et al., 2022). Whether artesunate can reduce PINP is completely unknown.

Activated excitatory glutaminergic receptors (Liu et al., 2014) and inhibitory gamma-aminobutyric acid receptor B (GABAB) (Kantamneni, 2015; Benke, 2022) and μ-opioid receptor (MOR) (Shueb et al., 2019) are major features of nociceptive synaptic plasticity in the entire nervous system. Long-term neuroinflammation is also an important mechanism of CINP (Lees et al., 1990). Paclitaxel induces the release of pro-inflammatory factors that exacerbate neuroinflammation and cause neuropathic pain, including tumor necrosis factor-α (TNF-α), interleukin-1β (IL-1β), and interleukin-6 (IL-6) (Ledeboer et al., 2007; Alé et al., 2014; Makker et al., 2017). Recent reports suggested that metabotropic glutamate receptor 5 (mGluR5) is involved in many pain models (Guo et al., 1991; Shueb et al., 2019; Wang et al., 2020) and regulates excitatory glutamate receptor trafficking and electrophysiological functions, which modulate *N*-methyl-D-aspartate (NMDA) receptors (Matta et al., 2011; Aloisi et al., 2017) and AMPAR receptors (She et al., 2009; Wan et al., 2011; Benneyworth et al., 2019) in neuronal transport on synapse membranes. We recently reported that artesunate impairs opioid-induced hyperalgesia by downregulating mGluR5 expression and reducing mitochondrial oxidative stress in the rat spinal cord. However, whether mGluR5 is involved in CINP remains undefined.

The current work revealed an important role for intrathecal artesunate therapy in mice with experimental PINP. In addition, the amounts of mGluR5, NMDA receptor 1 (NR1), α-amino-3-hydroxy-5-methyl-4-isoxazolepropionic acid (AMPA) receptor 2 (GluA2), GABAB, chemokines and pro-inflammatory factors in peripheral neurons were also assessed. Furthermore, mGluR5 agonist and antagonist were utilized for validating the antinociceptive mechanism of artesunate. The results suggest artesunate treatment may be a clinical approach to alleviate PINP.

## Experimental Procedures

### Mice

Eight-ten-week-old C57BL/6 J mice of both sexes were purchased from the Laboratory Animal Center of the Military Medical Science Academy of the Chinese People’s Liberation Army. Animal housing was carried out at 23 ± 2°C, with 50 ± 1% relative humidity under a 12-12 h light-dark cycle. Experiments involving animals had approval from the Tianjin Medical University institutional animal care committee and conformed with the AAALAC and IACUC guidelines (Tianjin, China; permit no. IRB2021-DW-16) as well as with the International Association for the Study of Pain. Mice underwent a 3-day adaptative housing prior to the study. All investigators were blinded to randomization and treatments. The number of animals in each group was 10, including 5 males and 5 females.

### Paclitaxel-Induced Neuropathic Pain Model

In brief, paclitaxel (TEVA Pharmaceuticals, Inc. United States) was dissolved in a 1:1 (v/v) mixture of Cremophor EL and absolute ethanol to 6 mg/ml and further diluted with 0.9% NaCl (normal saline) to 0.2 mg/ml immediately prior to dosing. The control vehicle was normal saline containing approximately 1.7% each of Cremophor EL and ethanol. Paclitaxel (4 mg/kg) or the vehicle control was intraperitoneally (i.p.) injected in the animals every other day at days 1, 3, 5, and 7, respectively (Mao et al., 2019; Qabazard et al., 2020).

### Drugs and Administration

Artesunate (MedChemExpress, China), 2-amino-2-(2-chloro-5-hydroxyphenyl) acetic acid (CHPG), 2-methyl-6-(phenyl ethynyl) pyridine (MPEP) and 10% dimethyl sulfoxide (DMSO as vehicle control) were administered, respectively, by intrathecal injection. The animals were anesthetized with sevoflurane (induction, 3.0%; maintenance, 1.5%) and drugs (5 μL) were administered intratracheally at the lumbar 4–5 level with 30-G needles (Zhang et al., 2022). The commercial drug’s information can be seen in Supplementary Data (Key Resources Table).

### Behavioral Tests

Mechanical stimuli, heat test and conditioned place preference (CPP) were carried out following previously reported protocols (Liang et al., 2016a,b; Sun et al., 2017; Zhao et al., 2017). Baseline threshold and latency were examined 1 day prior to drug administration, and animals were trained 2 h daily in testing apparatuses for 3 days prior to baseline testing. There was 1-h interval between the two tests. One investigator blinded to all treatments performed the totality of behavioral assays.

Paw withdrawal frequency (PWF) in response to mechanical stimuli was first measured. A 0.4-g von Frey filament (Stoelting Co., Wood Dale, IL, United States) was applied to the plantar surface of right hind paws. Stimulations were performed 10 times, and paw withdrawal response was presented as a percent response frequency [(number of paw withdrawals/10 trials) × 100].

Heat sensitivity was quantified in rodents by paw withdrawal latency (PWL), which was assessed with a Model 336 Analgesia Meter (IITC Inc. Life Science Instruments. Woodland Hills, CA, United States). In brief, the animals were individually placed in Plexiglass chambers on glass plates, through which a light beam was utilized for stimulating the plantar center. PWL was recorded as the time elapsed from the start of the light beam to foot withdrawal. Tests were carried out 5 times at 5-min intervals, for 20 s maximum to avoid tissue injury.

In the CPP assay, 3 daily 30-min preconditioning was firstly performed (MED Associates Inc.). On day 4, the time spent in every chamber was noted for totally 15 min. The conditioning assay was performed on days 5, 6, and 7, respectively, with closed internal door. The animals were intrathecally administered saline (5 μL) and placed in a conditioning chamber in the morning, followed by intrathecal administration of 5 μL lidocaine (0.4%) later in the afternoon and placement in another conditioning chamber, or vice versa. On day 8 (i.e., 10 days after the initial paclitaxel injection), each mouse was placed in a chamber with free access to both chambers, and the time spent in every chamber was noted for 15 min.

### Reverse Transcription-PCR

Quantitative real-time RT-PCR (qRT-PCR) was carried out following a previously reported protocol (Li et al., 2020). The mRNA levels of mGluR5, MOR, NR1, GluA2, and GABAB in the lumbar 3-5 (L3-5) segments of DRGs were determined. Total RNA extraction from DRG specimens utilized TRIzol reagent (Invitrogen, Grand Island, NY, United States). After treatment with excess DNase I (New England Biolabs, Ipswich, MA, United States), ThermoScript reverse transcriptase (Thermo Fisher Scientific) and oligo (dT) or specific RT-primers were utilized for cDNA synthesis. Template DNA underwent amplification by real-time PCR with primers described in Supplementary Data (Key Resources Table). Triplicate assays were run in 20 μL reactions containing forward and reverse primers (250 nM), SsoAdvanced Universal SYBR Green Supermix (10 μl; Bio-Rad, Hercules, CA, United States) and cDNA (20 ng). qRT-PCR was performed on a BIO-RAD CFX96 real-time PCR system. The 2^–Δ Δ *Ct*^ method was utilized for analysis, with GAPDH as a reference gene.

### Enzyme Linked Immunosorbent Assay

The DRGs tissues were mechanically homogenized in ice-cold lysis buffer (10 mM Tris, 2 mM MgCl2, 5 mM EGTA, 1 mM EDTA, 1 mM phenylmethylsulfonyl fluoride, 1 mM DTT, 40 μM leupeptin 40 μM leupeptin, and 250 mM sucrose). The lysate was centrifuged, and the supernatant was collected as the DRGs protein, and the protein concentrations of the samples were adjusted to 30 μg in the sample volume of 100 μl. The DRGs protein sample was used for quantifying chemokines [C-X-C motif chemokine ligand 1 (CXCL1), CXCL12 and C-C motif ligand 2 (CCL2)], inflammatory cytokines (TNF-α, IL-1β, and IL-6) and pain related receptors (mGluR5, MOR, NR1 GluA2, and GABAB) with commercially available ELISA kits as directed by the manufacturers. The lumbar DRGs of each two mice in the same group were combined into one sample, so the ELISA experiment of ten mice in each group was repeated five times. ELISA kits’ information can be seen in Supplementary Data (Key Resources Table).

### Statistical Analysis

The GraphPad Prism 9 software was utilized for data analysis. Box-and-whiskers plots were made, with each “box” reflecting the median and 25th and 75th quartiles and the corresponding “whisker” indicating the 5th and 95th percentiles. Behavioral data were compared by two-way repeated measures ANOVA, with *post-hoc* Tukey’s test for comparing multiple groups. qRT-PCR data were compared by one-way ANOVA with *post-hoc* Tukey’s test for multiple groups. ELISA data were compared by Mann–Whitney test or Kruskal–Wallis test for multiple groups. Sample size (*n* = 10) determination was performed based on similar behavioral and biochemical analyses (Li et al., 2020). *P* < 0.05 indicated a statistically significant difference.

## Results

### Paclitaxel-Induced Neuropathic Pain Is Accompanied by Metabotropic Glutamate Receptor 5 Upregulation in DRG

**FIGURE 1:**
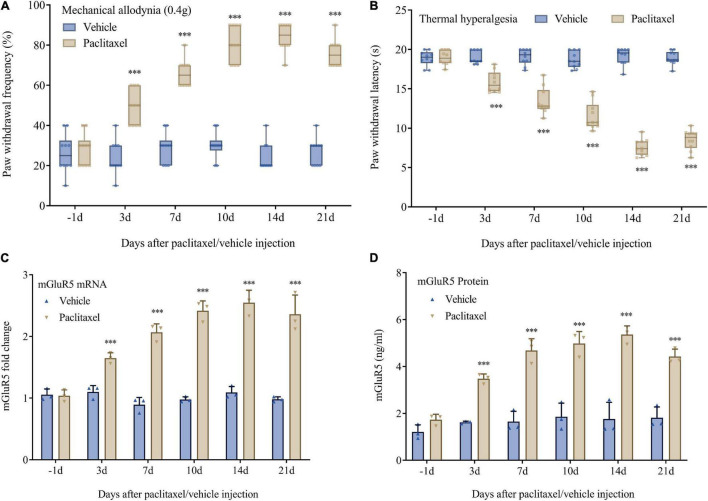
Paclitaxel-induced neuropathic pain is accompanied by upregulated DRG mGluR5 in mice. **(A)** Mechanical allodynia development as assessed by paw withdrawal frequency in the von Frey test. **(B)** Thermal pain development as assessed by paw withdrawal latency in the heat sensitivity test. DRG L3-5 segments were obtained for biochemical assays. **(C)** mGluR5 mRNA levels were determined as fold elevation vs. baseline values, with GAPDH utilized for normalization. **(D)** ELISA detection of mGluR5 levels after paclitaxel injection. Data are median with interquartile ranges, and individual data points are shown (*n* = 10). ***P < 0.001 vs. Vehicle group at the same time point.

Basal mechanical and heat sensitivity were comparable in both groups (*P* > 0.05, *n* = 10, Figures 1A,B). As previously reported (Luo X. et al., 2021), i.p. administration of paclitaxel, but not the vehicle, resulted in prolonged pain hypersensitivity (markedly increased PWF after stimulation with a 0.4 g von Frey filament) and heat hyperalgesia (starkly reduced PWL after heat stimulation) as shown in Figures 1A,B. Such effects started at 3 days following the initial paclitaxel treatment and continued for 3 weeks or more (Figures 1A,B). Paclitaxel injection also time-dependently elevated mGluR5 mRNA and protein amounts in DRG specimens. mGluR5 mRNA levels in L3-5 DRGs were increased on days 3, 7, 14, and 21 after the initial paclitaxel administration in comparison with vehicle-injected animals (Figure 1C). In agreement, mGluR5 protein amounts in L3-5 DRGs were increased on days 3, 7, 14, and 21 in comparison with the vehicle group (Figure 1D). As expected, the vehicle caused no changes in basal mGluR5 mRNA and protein amounts in DRGs (Figures 1C,D). The above data indicated paclitaxel injection promoted persistent DRG mGluR5 expression, which might be involved in PINP development and maintenance.

### Intrathecal Artesunate Administration Blunts Paclitaxel-Induced Neuropathic Pain

**FIGURE 2:**
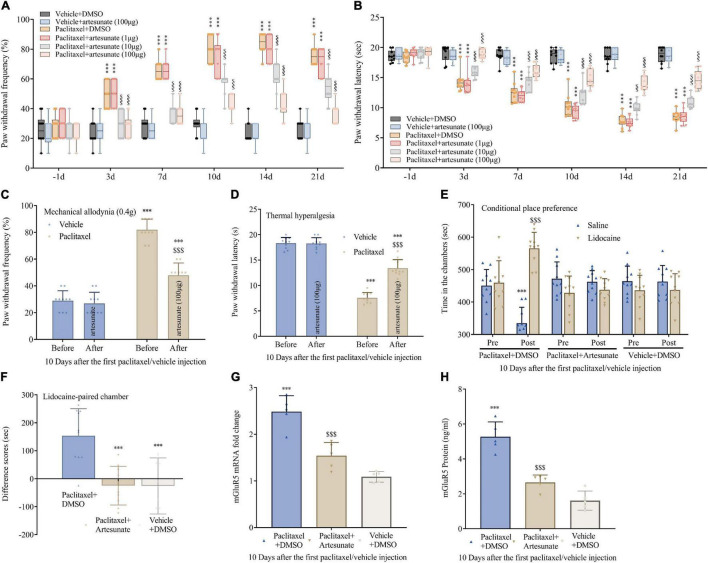
Pre- and post-treatments with artesunate decrease paclitaxel-induced neuropathic pain and DRG mGluR5 expression in mice. Artesunate was administered by intrathecal injection 10 min prior to paclitaxel injection. **(A,B)** Artesunate treatment starkly and dose-dependently decreased paclitaxel-associated mechanical allodynia and thermal pain. **(C,D)** A single administration of artesunate attenuated paclitaxel-induced mechanical paw withdrawal frequency and thermal paw withdrawal latency 10 days following the initial paclitaxel administration. **(E,F)** Effects of artesunate on paclitaxel-associated spontaneous ongoing pain based on conditional place preference. **(G,H)** qRT-PCR and ELISA identified the DRG levels of mGluR5 after paclitaxel injection and artesunate treatment. Data are median with interquartile ranges, and individual data points are shown (*n* = 10). **(A,B)** ***P < 0.001 vs. Vehicle + DMSO group; ^$$$^*P* < 0.001 vs. paclitaxel + DMSO group; **(F–H)** ***P < 0.001 vs. paclitaxel + DMSO group.

To assess artesunate’s specific effect on PINP, intrathecal administration of artesunate (1, 10, and 100 μg) was carried out 10 min before each paclitaxel injection. First, artesunate at 100 μg exerted no overt effects on peripheral mechanical and heat sensitivity compared to normal saline (Figures 2A,B). Interestingly, mice pretreated with artesunate at 10 and 100 μg, but not 1 μg, had decreased paclitaxel-induced mechanical allodynia and thermal hyperalgesia, reflected by starkly reduced PWF (Figure 2A) and remarkably increased PWL (Figure 2B). These anti-hyperalgesic effects of artesunate were pronounced at 3 days and continued for 21 days after the first paclitaxel injection, dose-dependently (Figures 2A,B). Additionally, we administered artesunate at 100 μg intrathecally on day 10 following the initial paclitaxel dosing and found that paclitaxel-induced mechanical and thermal pain were reduced (Figures 2C,D). In addition, 10 days following the initial paclitaxel administration, intrathecal treatment with artesunate at 100 μg alleviated paclitaxel-induced and stimulation-independent spontaneous ongoing pain, as reflected by no overt preference for the saline- or lidocaine-paired chamber in mice administered artesunate and paclitaxel. This contrasted the data obtained for paclitaxel-induced spontaneous ongoing pain, with an overt preference (increased time spent) for the lidocaine-paired chamber in mice administered DMSO and paclitaxel (Figures 2E,F). This suggests artesunate not only prevented paclitaxel-induced neuropathic mechanical and thermal pain, but also decreased pain after its occurrence, while also reducing spontaneous pain in mice.

### Artesunate Inhibits Paclitaxel-Induced Metabotropic Glutamate Receptor 5 Upregulation in DRGs

To explore whether mGluR5 contributes to the anti-nociceptive effect of artesunate, DRG mGluR5 amounts were examined after paclitaxel exposure. Surprisingly, pretreatment with artesunate suppressed DRG mGluR5 upregulation (Figures 2G,H). The above data indicated artesunate impaired the generation and maintenance of PINP *via* mGluR5 downregulation.

### Metabotropic Glutamate Receptor 5 Inhibition Reduces Paclitaxel-Induced Neuropathic Pain

**FIGURE 3:**
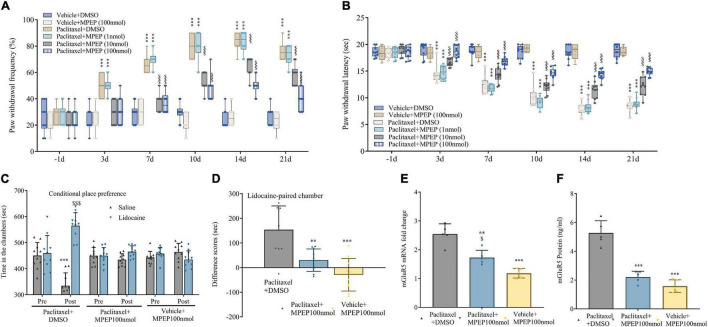
Paclitaxel-induced neuropathic pain (PINP) is relieved by the mGluR5 antagonist MPEP in mice. The mGluR5 antagonist MPEP was administered by intrathecal injection 10 min prior to paclitaxel treatment. **(A,B)** MPEP starkly and dose-dependently decreased paclitaxel-associated mechanical allodynia and thermal pain. **(C,D)** Treatment effects of the mGluR5 antagonist MPEP on paclitaxel-associated spontaneous ongoing pain based on conditional place preference. **(E,F)** qRT-PCR and ELISA identified the DRG levels of mGluR5 after paclitaxel injection and MPEP treatment. Data are median with interquartile ranges, and individual data points are shown (*n* = 10). **(A,B)** ***P < 0.001 vs. Vehicle + DMSO group; ^$$$^*P* < 0.001 vs. paclitaxel + DMSO group; **(D–F)** **P < 0.01, ***P < 0.001 vs. paclitaxel + DMSO group; ^$^*P* < 0.05 vs. Vehicle + MPEP 100 nmol group.

To confirm mGluR5’s effect in transducing nociception in PINP, mice were treated by intrathecal injection of MPEP (mGluR5 antagonist; 1, 10, or 100 nmol) 10 min before every paclitaxel injection. As anticipated, behavioral assays showed that mechanical allodynia and thermal hyperalgesia were robustly and dose-dependently reversed by MPEP at 10 and 100 nmol, but not 1 nmol, as reflected by drastically increased paw withdrawal mechanical threshold and thermal latency in mice administered paclitaxel (Figures 3A,B). The anti-allodynic activity of MPEP was pronounced at 3 days and persisted for 3 weeks. However, MPEP at 100 nmol had no effects on basal mechanical and heat sensitivity in comparison to control mice (Figures 3A,B). In addition, 10 days following the initial paclitaxel dosing, intrathecal treatment with MPEP at 100 nmol alleviated paclitaxel-induced and stimulation-independent spontaneous ongoing pain, as reflected by no overt preference for the saline- or lidocaine-paired chamber in mice administered artesunate and paclitaxel. The above finding contrasted data obtained with paclitaxel-induced spontaneous ongoing pain as reflected by an overt preference (increased time spent) for the lidocaine-paired chamber in mice administered DMSO and paclitaxel (Figures 3C,D). DRG mGluR5 amounts were examined after paclitaxel exposure, and pretreatment with MPEP 100 nmol suppressed DRG mGluR5 upregulation by paclitaxel (Figures 3E,F). This suggests MPEP prevented paclitaxel-induced neuropathic mechanical and thermal pain, while also reducing spontaneous pain. Jointly, the above findings demonstrated the important role of DRG mGluR5 in PINP in mice.

### Combination of the Metabotropic Glutamate Receptor 5 Antagonist 2-Methyl-6-(Phenyl Ethynyl) Pyridine and Artesunate Has a Stronger Anti-hyperalgesic Effect Than Artesunate Alone in the Paclitaxel-Induced Neuropathic Model

**FIGURE 4:**
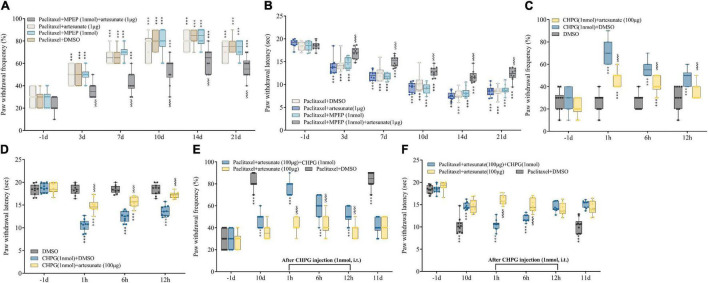
Involvement of mGluR5 in artesunate antinociception in PINP in mice. **(A,B)** Artesunate (1 μg) and the mGluR5 antagonist MPEP (1 nmol) were administered by intrathecal injection 10 min prior to paclitaxel treatment. Artesunate and MPEP jointly administered at subthreshold amounts starkly reduced paclitaxel-associated mechanical allodynia and thermal pain. **(C,D)** Artesunate (100 μg) administration was performed by injection 10 min prior to treatment with the mGluR5 agonist CHPG (1 nmol). Artesunate treatment starkly reduced CHPG-associated mechanical allodynia and thermal hyperalgesia. **(E,F)** CHPG injection (1 nmol) was administered to mice 10 days after the initial paclitaxel administration. The pain alleviating effect of artesunate on PINP was partially reversed by intrathecal injection of the mGluR5 agonist CHPG. Data are median with interquartile ranges, and individual data points are shown (*n* = 10). ***P < 0.001 vs. Baseline (−1d); ^$$$^*P* < 0.001 vs. Paclitaxel + DMSO group/DHPG + DMSO group/Paclitaxel + artesunate + GHPG group at the same time point.

Next, von Frey and hot plate assays demonstrated artesunate (1 μg) and MPEP (1 nmol) at subthreshold doses had no effects on paw withdrawal threshold and latency when administered alone (Figures 3E,F). However, mechanical allodynia and thermal hyperalgesia were efficiently controlled by joint administration of artesunate and MPEP (Figures 4A,B). The above findings further indicated mGluR5 was involved in the anti-PINP activity of artesunate.

### Acute Pain Triggered by the Metabotropic Glutamate Receptor 5 Agonist 2-Amino-2- (2-Chloro-5-Hydroxyphenyl) Acetic Acid Is Attenuated by Artesunate Therapy

Next, whether artesunate also alleviates acute pain triggered by intrathecal treatment with the mGluR5 agonist CHPG at 1 nmol was examined. Artesunate (100 μg) administration was performed by intrathecal injection 10 min prior to CHPG treatment. Our results revealed CHPG induced acute mechanical allodynia and thermal hyperalgesia in naïve animals from 1 to 12 h after intrathecal dosing (Figures 4C,D). Interestingly, artesunate treatment sufficiently prevented CHPG-associated acute pain (Figures 4C,D). Collectively, the above data further indicated the important role of mGluR5 in chemotherapy-related pain and artesunate-associated anti-nociception.

### The Alleviating Effect of Artesunate on Paclitaxel-Induced Neuropathic Pain Can Be Partially Reversed by Intrathecal Injection of the Metabotropic Glutamate Receptor 5 Agonist 2-Amino-2-(2-Chloro-5-Hydroxyphenyl) Acetic Acid

We have demonstrated the therapeutic effect of artesunate on paclitaxel-induced chemotherapy-triggered pain in mice. Next, we wondered whether temporary administration of CHPG would reduce the anti-hyperalgesic effect of artesunate. CHPG injection (intrathecal, 1 nmol) was administered to mice that developed chemotherapy-related pain 10 days following the initial paclitaxel dosing. We found that the anti-hyperalgesic effects of artesunate were partially antagonized by CHPG injection, including increased mechanical and thermal pain (Figures 4E,F). These findings suggested artesunate’s anti-hyperalgesic effect on chemotherapy-induced pain is not solely dependent on mGluR5, and other anti-hyperalgesic mechanisms may exist. Therefore, we conducted subsequent experiments, focusing on known pain-related proteins, chemokines and pro-inflammatory factors in paclitaxel-induced neuropathy, to explore whether other signaling pathways are involved in artesunate-mediated relief of chemotherapy-based pain.

### Artesunate Regulates *N*-Methyl-D-Aspartate (NMDA) Receptor 1 and α-Amino-3-Hydroxy-5-Methyl-4-Isoxazolepropionic Acid (AMPA) Receptor 2 in DRGs *via* Metabotropic Glutamate Receptor 5

**FIGURE 5:**
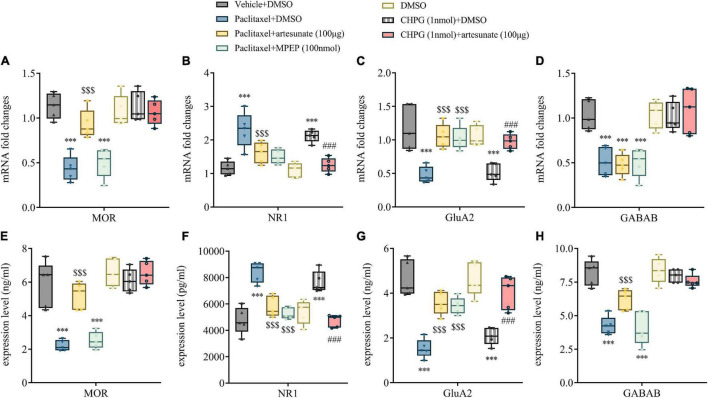
Artesunate alleviates PINP by regulating NR1 and GluA2 in DRGs *via* mGluR5 in mice. Bilateral L3-5 DRGs were obtained from mice 10 days following the initial paclitaxel treatment. qRT-PCR detected MOR **(A)**, NR1 **(B)**, GluA2 **(C)**, and GABAB **(D)** mRNA expression levels in DRGs, expressed as fold elevation vs. Vehicle + DMSO group, with GAPDH utilized as a reference. ELISA detection of the DRG levels of MOR **(E)**, NR1 **(F)**, GluA2 **(G)**, and GABAB **(H)** in mice. Data are median with interquartile ranges, and individual data points are shown (*n* = 5). ***P < 0.001 vs. Vehicle + DMSO group; ^$$$^*P* < 0.001 vs. Paclitaxel + DMSO group; ^###^*P* < 0.001 vs. CHPG (1 nmol) + DMSO group.

Bilateral L3-5 DRGs were obtained from mice 10 days following the initial paclitaxel administration, and the mRNA and membrane protein expression levels of receptors clearly associated with pain, including MOR, NR1, GluA2, and GABAB, were examined. We found NR1 upregulation in DRG specimens from paclitaxel-treated mice, while MOR, GluA2, GABAB receptor levels were decreased. Compared with the paclitaxel group, NR1 expression was decreased and GluA2 expression was increased in paclitaxel + artesunate-treated and paclitaxel + MPEP-treated DRGs. CHPG treatment increased NR1 expression and decreased GluA2 amounts in DRG, similar to paclitaxel. However, both CHPG and MPEP had no effects on MOR and GABAB expression levels (Figures 5A–H). Artesunate exerted an anti-hyperalgesic effect through mGluR5 regulation of NR1 and GluA2 in the PINP model.

### Artesunate Relieves Paclitaxel-Induced Neuroinflammation

**FIGURE 6:**
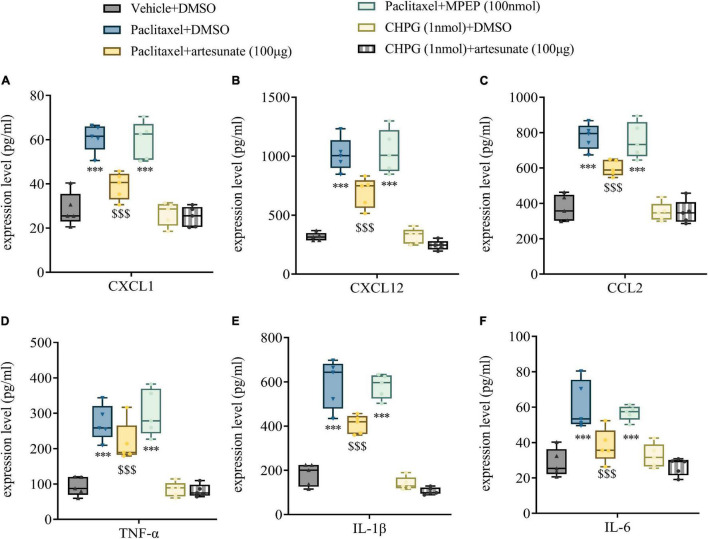
Artesunate reduces DRG neuroinflammation by paclitaxel treatment in mice. Bilateral L3-5 DRGs were obtained from mice 10 days following the initial paclitaxel administration. ELISA detected the DRG levels of CXCL1 **(A)**, CXCL12 **(B)**, CCL2 **(C)**, TNF-α **(D)**, IL-1β **(E)**, and IL-6 **(F)** in mice. Data are median with interquartile ranges, and individual data points are shown (*n* = 5). ***P < 0.001 vs. Vehicle + DMSO group; ^$$$^*P* < 0.001 vs. Paclitaxel + DMSO group.

Artesunate has a strong anti-inflammatory effect, which might be involved in the anti-hyperalgesic mechanism of PINP. However, whether mGluR5 is involved in the anti-inflammatory mechanism of artesunate still needs to be determined. L3-5 DRGs were harvested 10 days following the initial paclitaxel treatment, and chemokines and pro-inflammatory cytokines related to paclitaxel injection were quantitated. We found that the amounts of chemokines (CXCL1, CXCL12, and CCL2) and pro-inflammatory cytokines (TNF-α, IL-1β, and IL-6) were significantly increased in DRGs treated with paclitaxel, while the mGluR5 agonist CHPG had no significant effects on CXCL1, CXCL12, CCL2, TNF-α, IL-1β, and IL-6 amounts. Artesunate reduced the increased expression levels of CXCL1, CXCL12, CCL2, TNF-α, IL-1β, and IL-6 observed after paclitaxel treatment. However, the mGluR5 antagonist MPEP did not significantly affect paclitaxel-induced increases in chemokines and pro-inflammatory cytokines. This finding suggested that artesunate could reduce PINP through an anti-inflammatory mechanism, while mGluR5 is not involved in the anti-inflammatory mechanism of artesunate (Figures 6A–F).

## Discussion

This study identified previously unrecognized effects of artesunate in the prevention and treatment of PINP *via* suppression of mGluR5 activation and neuroinflammation. Specifically, paclitaxel-associated mechanical allodynia and thermal pain as well as spontaneous pain in the animals were evident from day 3 to at least 21 days after paclitaxel injection, which was accompanied by increased levels of mGluR5, chemokines and pro-inflammatory factors in DRGs. Furthermore, artesunate treatment dose-dependently prevented PINP symptoms and alleviated DRG mGluR5, CXCL1, CXCL12, CCL2, TNF-α, IL-1β, and IL-6 elevations. Meanwhile, intrathecal antagonism of mGluR5 ameliorated the PINP phenomenon, and the expression levels of NR1 and GluA2 were specifically altered. Finally, intrathecal injection of CHPG directly caused transient pain and altered the expression of NR1 and GluA2, which were reversed by artesunate. These results suggest that artesunate may act against PINP by altering mGluR5-mediated changes in NR1 and GluA2 expression and reducing neuroinflammation.

A growing number of reports have demonstrated the contribution of mGluR5-induced central nervous system (CNS) neurotoxicity to the development of diverse neuropsychiatric diseases. More recently, peripheral inflammatory reactions and neurotrauma have been shown to activate mGluR5 at excitatory nociceptive synapses, resulting in acute inflammatory pain and prolonged neuropathic allodynia (Vincent et al., 2017; Lai et al., 2018). In addition, mGluR5-dependent synaptic plasticity in opioid-triggered analgesic tolerance is regulated in animal models (Zhou et al., 2013). Here, we report for the first time a rapid increase in mGluR5 expression in the DRG of mice administered paclitaxel, which corroborates the course of the PINP phenotype. The above evidence highly indicates DRG mGluR5 activation in the pathophysiological process of PINP. Nonetheless, the mechanism by which mGluR5 controls DRG nociception after paclitaxel administration requires further investigation.

In addition to mGluR5, NMDAR, and AMPAR are co-expressed in neuronal synapses (Goncalves et al., 2020). mGluR5 modulates the expression of glutamate receptors [NMDA (Bertaso et al., 2010; Dai et al., 2014; Aloisi et al., 2017) and AMPA receptors (Huber et al., 2002)] in neurons, suggesting its potential involvement in neuropathic pain. Paclitaxel-induced neuropathy involves elevated presynaptic mGluR5 activity in the spine, serving as an upstream signal for PKC-induced tonic increase of NMDARs (Xie et al., 2017). Chemotherapy-associated tonic activation of presynaptic NMDARs promotes glutamate release at synapses in the spinal dorsal horn (Xie et al., 2016). Both Group I mGluRs and NMDARs highly contribute to maintaining prolonged elevation of excitatory synaptic transmission (Yashpal et al., 2001; Yasuda et al., 2018). Therefore, administration of ketamine can significantly relieve PINP and reduce the consumption of opioids (Pascual et al., 2010). mGluR5-dependent decrease of GluA2-expressing AMPARs at NAc shell synapses receiving signals from the infralimbic cortex represents an important inducing factor in reinstating the cocaine-primed conditioned approach behavior (Benneyworth et al., 2019). mGluR5 activation increases neuronal NR1 expression and GluA2 internalization (Benneyworth et al., 2019), thereby increasing NMDAR- and AMPAR-mediated neuronal excitatory afferents. Additionally, μ-opioid receptors in primary afferent nociceptors induce and sustain opioid-associated tolerance, hyperalgesia and pre-sensory synaptic plasticity (Corder et al., 2017). The expression level of MOR is decreased in the spinal nerve ligation neuropathic pain state (Wu et al., 2019). Therefore, MMG22, a dual-acting MOR agonist and mGluR5 antagonist, has significant anti-hyperalgesic effects, especially in cancer pain, inflammatory pain and neuropathic pain (Akgün et al., 2013; Shueb et al., 2019; Speltz et al., 2020). GABAARs are the primary determinants of rapid synaptic inhibition in the CNS. Kiss et al. (2021) showed cerebral gephyrin and γ2-GABAA-receptor protein amounts are rescued in mice jointly administered artemisinin and artesunate, with most pronounced effects at a low artesunate dose. Artemisinins decease inhibitory neurotransmission with an obligatory dependence on gephyrin; however, the ability of artesunate to decrease the neuron transmission effect of GABAARs is lowest (Kasaragod et al., 2019). The pain-related signaling molecules and pathways involved in the anti-hyperalgesic effect of artesunate are very complex. So far, we have found NR1 and GluA2 are involved, but the specific signaling pathway still needs to be further explored.

Chemotherapy-induced peripheral neurotoxicity (CIPN) involves mechanical allodynia, abnormal neuronal responses and dysregulated pro- and anti-inflammatory factors in the spinal dorsal horn. Neuroinflammation is one of the most critical mechanisms of paclitaxel-mediated neuropathic pain development (Meesawatsom et al., 2020). The chemokines CXCL1, CXCL12, and CCL2 have been shown to contribute to the immune reactions after paclitaxel treatment (Pevida et al., 2013; Xu et al., 2018; Manjavachi et al., 2019). Artemisinin and derived compounds show powerful anti-inflammatory, antioxidant and neuroprotective activities (Kim et al., 2015; Qiang et al., 2018; Guan et al., 2021; Ahmed-Laloui et al., 2022). Artemisinin inhibits NO secretion by LPS-treated BV2 cells and markedly downregulates the inflammatory factors IL-1β, IL-6, and TNF-α (Qiang et al., 2018). However, the anti-inflammatory effect of artemisinin on chemokines has not been reported. The powerful anti-inflammatory effect of artesunate was shown in this study, with significantly decreased pro-inflammatory cytokine (IL-1β, IL-6, and TNF-α) and chemokine (CXCL1, CXCL12, and CCL2) expression levels. These findings clearly suggest artesunate could be applied for treating paclitaxel-induced neuroinflammation.

This research demonstrated the excellent therapeutic effects of artemisinin on chemotherapy pain and neuropathy. It is worth mentioning that artesunate has an affordable price, low toxicity and high efficiency, which may be beneficial to clinical relief of CINP. Remarkably, artemisinins efficiently cross the blood-brain barrier (Davis et al., 2003). Because artesunate shows regulatory effects on various pain-related ion channels, anti-neuroinflammatory activity and anti-cancer properties (Crespo-Ortiz and Wei, 2012), this drug may be very suitable for simultaneous use with other chemotherapeutic drugs to prevent the development of chemotherapy pain. It is also a good option to combine other analgesics as a component of multimodal analgesia, which can reduce opioid dosage and side effects. Of course, whether artesunate prevents pain in the clinical setting, and its impact on patient outcomes deserve further investigation.

One limitation of our current study is that we did not investigate how artesunate reduces mGluR5 in neuropathic pain conditions, which is consistent with our previous report in a rodent model of remifentanil-induced hyperalgesia (Zhang et al., 2022). After checking with literature, it was suggested that mTOR may be involved in the modulation of mGluR5 by artesunate. Artesunate and dihydroartemisinin (DHA) can induce the regulation-related proteins of mTOR (raptor) to dissociate from mTOR and inhibit mTORC1 activity (Luo J. et al., 2021). Artesunate protects against ischemic cerebral infarction, which is associated with regulating the activity of mTOR by decreasing p-mTOR level (Shao et al., 2018). In addition, artesunate or the mTOR inhibitor rapamycin (Rapa) can inhibit the harmful signaling pathways HMGB1/RAGE and TLR4/MyD88/TRAF6 to inactivate the transcription factor NF-κB and reduce its pro-inflammatory cytokines (IL-1β, IL-18, IL-6, and TNF-α), thereby alleviating liver ischemia-reperfusion injury (Ghoneim et al., 2020), suggesting that artesunate may exert an anti-inflammatory effect through mTOR. Simultaneously, mTOR is required for the activation of mGluR5. Down-regulation of mGluR5 in a rat model of migraine activates autophagy by inhibiting the mTOR pathway and reduces the expression of central sensitization-related proteins CGRP and SP (Niu et al., 2021). We suspect that the mTOR pathway may be a key cascade that mediates the analgesic effect of artesunate by inhibiting mGluR5 and the anti-inflammatory effect of artesunate, which needs to be confirmed in future experiments.

Nerve injury is accompanied by chronic inflammation, leading to the generation and maintenance of neuropathic pain. Inflammatory mediators such as secreted phospholipase A2 (sPLA2) regulate nociceptive and excitatory neuronal signaling during the development of pain through hydrolytic activity (Kartha et al., 2021). In the chemotherapy pain model, it was demonstrated that the pro-inflammatory mediators in the dorsal root ganglion (DRG) increased, indicating the induction of neuroinflammation in DRG. It is well known that neuroinflammation in glial cells can increase the release of presynaptic glutamate, thereby activating postsynaptic mGluR5 in trigeminal neuralgia (Honda et al., 2017). The increase of mGluR5 and GluR1 expression in the spinal cord in a painful nerve root compression model may induce presynaptic neurotransmitter glutamate release, whereas glutamate clearance impairs mGluR5 over-expression and neuropathic pain-like behaviors. Balancing glutamate release and uptake after nerve injury should be an important goal in the treatment of chronic neuropathic pain (Inquimbert et al., 2012). Although this present study did not monitor glutamate levels within the DRG, which is one of our limitations, we speculate that neuroinflammation may cause the release of glutamate to activate mGluR5 in the pathogenesis of chronic pain, and artesunate exerts positive anti-inflammatory effects to reduce the activation of mGluR5 to further achieve anti-inflammatory and analgesic effects.

In addition, we found a very interesting phenomenon—a strong upregulation of Myelin protein zero (MPZ) by artesunate (data not shown). Simple administration of artesunate could significantly enhance the expression of MPZ. Artemisinin is suspected to significantly upregulate MPZ in DRG; because MPZ is an important protein in Schwann cells, it is critical for the formation of myelin and the maintenance of normal morphology (Torii et al., 2019; Moss et al., 2021). In addition, demyelination is an important mechanism of paclitaxel-induced neuropathy (Moscarello et al., 2002). Artemisinin can obviously reverse paclitaxel-induced MPZ decrease, suggesting that artemisinin may exert the effect of reducing nerve demyelination. We are very interested in this phenomenon, which is being further examined by our team.

In conclusion, artesunate can effectively prevent and treat PINP, possibly by inhibiting mGluR5-related neuroplasticity and reducing neuroinflammation. The present results indicate artesunate represents a novel therapeutic option in neuropathic pain after chemotherapy.

## Data Availability Statement

The original contributions presented in the study are included in the article/Supplementary Material, further inquiries can be directed to the corresponding authors.

## Ethics Statement

The animal study was reviewed and approved by the Tianjin Medical University Institutional Animal Care Committee.

## Author Contributions

LZ and JY conceived the experiment. YL, JK, CXW and YX collected the data. NL, JY, and CYW analyzed the data. YL, YY, and GW wrote the manuscript. All authors contributed to the article and approved the submitted version.

## Conflict of Interest

The authors declare that the research was conducted in the absence of any commercial or financial relationships that could be construed as a potential conflict of interest.

## Publisher’s Note

All claims expressed in this article are solely those of the authors and do not necessarily represent those of their affiliated organizations, or those of the publisher, the editors and the reviewers. Any product that may be evaluated in this article, or claim that may be made by its manufacturer, is not guaranteed or endorsed by the publisher.

## Abbreviations

CIPN: chemotherapy-induced peripheral neurotoxicity
PINP: paclitaxel-induced neuropathic pain
DMSO: dimethyl sulfoxide
GAPDH: glyceraldehyde 3-phosphate dehydrogenase
CHPG: 2-amino-2-(2-chloro-5-hydroxyphenyl) acetic acid
MPEP: 2-methyl-6-(phenyl ethynyl) pyridine
mGluR5: metabotropic glutamate receptor 5
MOR: μ-opioid receptor
NR1: *N*-methyl -D-aspartate (NMDA) receptor 1
GluA2: α-amino -3-hydroxy-5-methyl-4-isoxazolepropionic acid (AMPA) receptor 2
GABAB: Gamma-Aminobutyric Acid receptor B
CXCL1: C-X -C motif chemokine ligand 1
CXCL12: C-X -C motif chemokine ligand 12
CCL2: chemokine (C-C motif) ligand 2
TNF-α: tumor necrosis factor-α
IL-1β: interleukin-1β
IL-6: interleukin-6.
