# Supplementary figures and images for: Artesunate Alleviates Paclitaxel-Induced Neuropathic Pain in Mice by Decreasing Metabotropic Glutamate Receptor 5 Activity and Neuroinflammation in Primary Sensory Neurons
Source: Front Mol Neurosci. 2022 May 27;15:902572. doi: 10.3389/fnmol.2022.902572 (PMC9184756; doi:10.3389/fnmol.2022.902572)

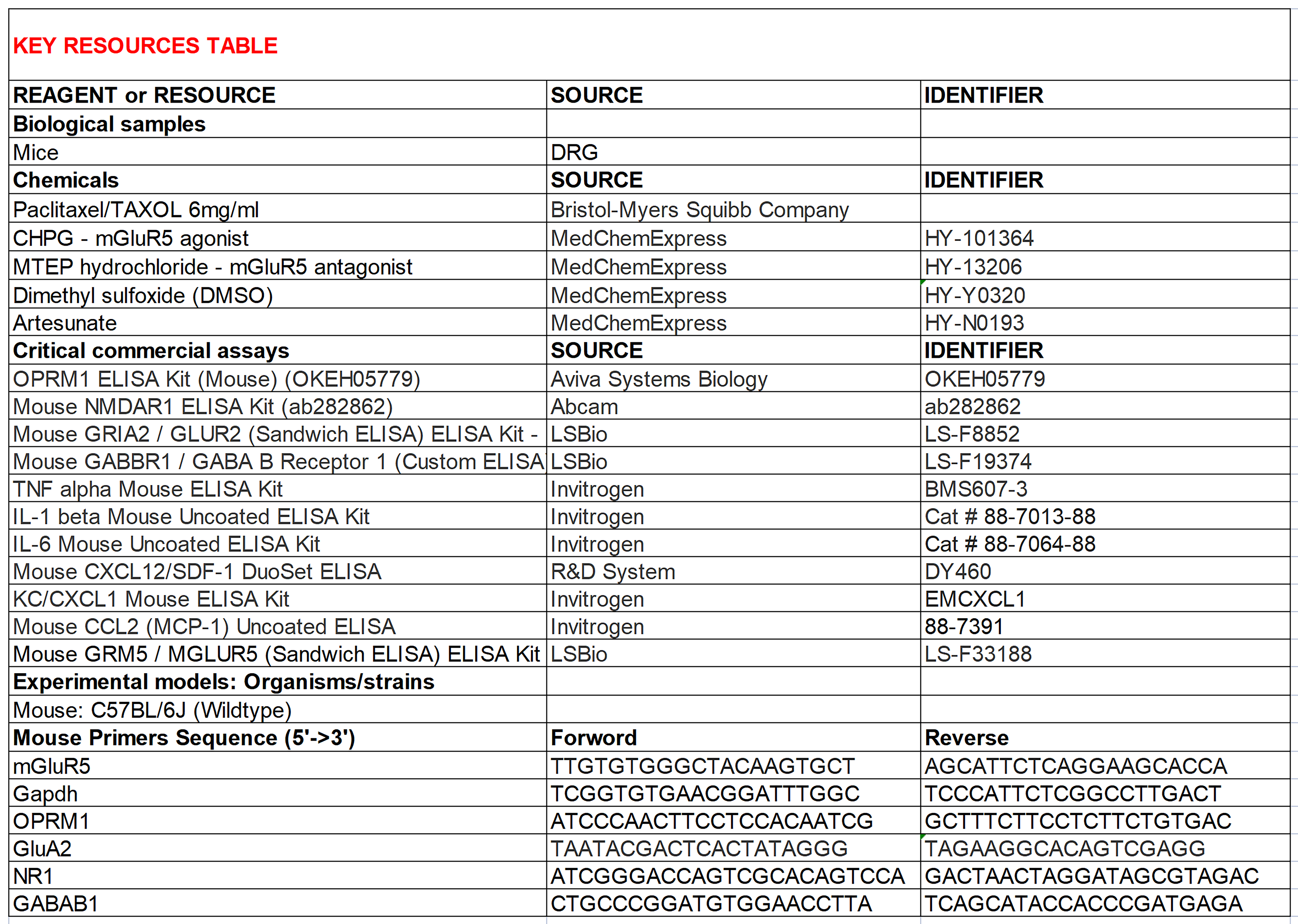

Supplement: Supplementary file 7 [file Image_1.TIF]
